# Supplementary material for: From sequence to enzyme mechanism using multi-label machine learning
Source: BMC Bioinformatics. 2014 May 19;15:150. doi: 10.1186/1471-2105-15-150 (PMC4229970; doi:10.1186/1471-2105-15-150)
Supplement: Additional file 2 — Java code of ml2db. Additional file ml2db_code.tar.gz contains the Java source code to run the multi-label machine learning experiments and save the results to database. The code’s Javadoc is included. [file 1471-2105-15-150-S2.zip › additional file 2/ml2db/ml2db/doc/main/ml2db/mulan/learn/MulanSerializer.html]

MulanSerializer


JavaScript is disabled on your browser.


- Overview
- Package
- Class
- Use
- Tree
- Deprecated
- Index
- Help

- Prev Class
- Next Class

- Frames
- No Frames

- All Classes

- Summary:
- Nested |
- Field |
- Constr |
- Method

- Detail:
- Field |
- Constr |
- Method


main.ml2db.mulan.learn

## Class MulanSerializer

- java.lang.Object
- - main.ml2db.Serializer
  - - main.ml2db.mulan.learn.MulanSerializer

- ---

    

  ```
  public class MulanSerializer
  extends Serializer
  ```

  Takes an arff file and an algorithm. Builds the trained learner. Saves the
  trained classifier to file as a serialized object ('.model' suffix).

  Version:
  :   29 Jun 2010

  Author:
  :   Luna De Ferrari ldf(at)st-andrews.ac.uk

- - ### Constructor Summary

    Constructors

    Constructor and Description

    `MulanSerializer(MulanArffRecord arffRecord,
    MulanLearner learner,
    java.lang.String directoryForSerializedModel)`
  - ### Method Summary

    Methods

    | Modifier and Type | Method and Description |
    | --- | --- |
    | `java.lang.String` | `buildAndSerialize()` |
    | `static mulan.classifier.MultiLabelLearnerBase` | `deserialize(java.lang.String filePath)` Get trained learner from file |
    | `MulanArffRecord` | `getArffRecord()` |
    | `MulanLearner` | `getLearner()` |
    | `static void` | `main(java.lang.String[] args)` |
    | `static java.lang.String` | `run(int trainArffId, MulanLearner learner, DbManagers dbMan, java.lang.String folderForModel)` |
    | `java.lang.String` | `serialize(mulan.classifier.MultiLabelLearner model)` |

    - ### Methods inherited from class main.ml2db.Serializer

      `clone, getBaseFileName, getDirectoryForSerializedObject, getFileForSerializedObject, getLogFile, serialize, setFileForSerializedModel, setLogFile`
    - ### Methods inherited from class java.lang.Object

      `equals, getClass, hashCode, notify, notifyAll, toString, wait, wait, wait`

- - ### Constructor Detail


    - #### MulanSerializer

      ```
      public MulanSerializer(MulanArffRecord arffRecord,
                     MulanLearner learner,
                     java.lang.String directoryForSerializedModel)
      ```
  - ### Method Detail


    - #### buildAndSerialize

      ```
      public java.lang.String buildAndSerialize()
      ```

      Returns:
      :   the path to the serialised model


    - #### getArffRecord

      ```
      public MulanArffRecord getArffRecord()
      ```


    - #### getLearner

      ```
      public MulanLearner getLearner()
      ```


    - #### serialize

      ```
      public java.lang.String serialize(mulan.classifier.MultiLabelLearner model)
      ```


    - #### deserialize

      ```
      public static mulan.classifier.MultiLabelLearnerBase deserialize(java.lang.String filePath)
      ```

      Get trained learner from file

      Parameters:
      :   `filePath` - the full file path

      Returns:
      :   a multi-label learner base


    - #### main

      ```
      public static void main(java.lang.String[] args)
      ```


    - #### run

      ```
      public static java.lang.String run(int trainArffId,
                         MulanLearner learner,
                         DbManagers dbMan,
                         java.lang.String folderForModel)
      ```


- Overview
- Package
- Class
- Use
- Tree
- Deprecated
- Index
- Help

- Prev Class
- Next Class

- Frames
- No Frames

- All Classes

- Summary:
- Nested |
- Field |
- Constr |
- Method

- Detail:
- Field |
- Constr |
- Method
